# A High Sensitivity Electric Field Microsensor Based on Torsional Resonance

**DOI:** 10.3390/s18010286

**Published:** 2018-01-19

**Authors:** Zhaozhi Chu, Chunrong Peng, Ren Ren, Biyun Ling, Zhouwei Zhang, Hucheng Lei, Shanhong Xia

**Affiliations:** 1 State Key Laboratory of Transducer Technology, Institute of Electronics, Chinese Academy of Sciences, Beijing 100190, China; czz_casie@163.com (Z.C.); crpeng@mail.ie.ac.cn (C.P.); renren@mail.ie.ac.cn (R.R.); lingbiyun15@mails.ucas.ac.cn (B.L.); zhangzhouwei15@mails.ucas.ac.cn (Z.Z.); leihucheng16@mails.ucas.ac.cn (H.L.); 2 University of Chinese Academy of Sciences, Beijing 100039, China

**Keywords:** electric field microsensor, torsional resonance, MEMS, efficiency of charge induction

## Abstract

This paper proposes a high sensitivity electric field microsensor (EFM) based on torsional resonance. The proposed microsensor adopts torsional shutter, which is composed of shielding electrodes and torsional beams. The movable shielding electrodes and the fixed sensing electrodes are fabricated on the same plane and interdigitally arranged. Push–pull electrostatic actuation method is employed to excite the torsional shutter. Simulation results proved that the torsional shutter has higher efficiency of charge induction. The optimization of structure parameters was conducted to improve its efficiency of charge induction further. A micromachining fabrication process was developed to fabricate the EFM. Experiments were conducted to characterize the EFM. A good linearity of 0.15% was achieved within an electrostatic field range of 0–50 kV/m, and the uncertainty was below 0.38% in the three roundtrip measurements. A high sensitivity of 4.82 mV/(kV/m) was achieved with the trans-resistance of 100 MΩ, which is improved by at least one order of magnitude compared with previously reported EFMs. The efficiency of charge induction for this microsensor reached 48.19 pA/(kV/m).

## 1. Introduction

The measurement of electrostatic field strength is of great importance for aeronautics and astronautics, high voltage direct current (HVDC) power system, meteorology and so on [1,2,3,4,5,6,7,8]. For example, in the field of aeronautics and astronautics, the satellites cannot be launched unless the atmospheric electric field strength is below certain threshold, and many facts indicate that strong atmospheric electric field may cause launching failure. Thus, sensors that can accurately quantify the strength of the atmospheric electric field are in great demand. A typical type of sensors used in electrostatic field measurement are rotating-vane field mills based on charge induction. With electric motors shielding the electrostatic field periodically, alternating current with respect to strength of electrostatic field around the sensors is produced. However, rotating-vane field mills have disadvantages of big volumes, complex structures and high power consumption.

In the last few decades, with the development of Micro-electro-mechanical Systems (MEMS) technology, a variety of the electric field microsensors (EFMs) have been developed with advantages of small size, lower power consumption and low cost. Almost all the EFMs are also based on charge induction and designed to work in resonant modes [9,10,11,12,13,14,15,16,17,18,19,20,21]. In 1991, an electrostatic voltmeter based on MEMS technology was firstly proposed by Hsu [9]. The microsensor works on the principle of intermittent shuttering and exposing a sensing electrode to an electric field. In 2006, Peng developed a micromechanical resonant electrostatic field sensor, using lateral electrostatic actuation [11]. However, as for these two microsensors, the sensing electrodes are patterned on the substrate beneath the shielding electrodes. Due to the fringe effect of the electric field, most components of the measured electric field distribute on the shielding electrodes rather than the sensing electrodes, which leads to low sensitivity of the EFMs.

Aimed at increasing the sensitivity of EFMs, in 2003, an EFM with lateral vibration and coplanar electrodes was proposed by Riehl [13], wherein the fringe effect of the electric field on the sidewall was utilized to improve the efficiency of charge induction. Moreover, to further improve the sensitivity, Yang developed an SOI-based electric field sensor with comb-shaped microelectrodes [15]. The sensitivity of this microsensor was about 0.2 mV/(kV/m). However, these microsensors mainly modulated the electric field distributing on the sidewalls of the sensing electrodes. Since the component of the measured electric field distributing on the top surface of the sensing electrodes was almost not modulated, it is very likely that the sensitivity of EFMs can be improved by meticulous design.

In this paper, we proposed a high sensitivity EFM based on torsional resonance for electrostatic field measurement. Different from previous EFMs which utilized the lateral shutter, the proposed EFM adopted torsional shutter, which was composed of shielding electrodes and torsional beams. The movable shielding electrodes and the fixed sensing electrodes were fabricated on the same plane and interdigitally arranged. Push–pull electrostatic actuation method was employed to excite the torsional shutter. The optimization of structure parameters was conducted to improve its charge induction efficiency further. A micromachining fabrication process was developed to fabricate the EFM.

## 2. Working Principle and Structure Design

### 2.1. Working Principle

**Figure 1:**
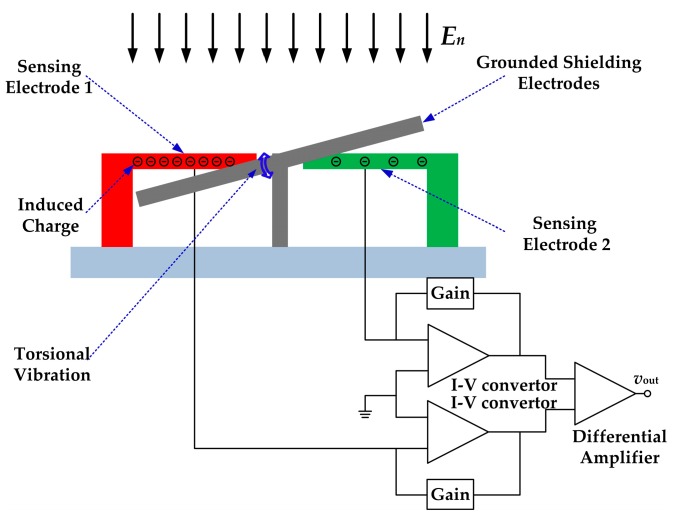
The working principle of the electric field microsensor (EFM) with torsional shutter.

The working principle of the microsensor is shown in Figure 1. Two identical arrays of sensing electrodes are symmetrically located on the substrate, and the grounded shielding electrodes are arranged along their axis of symmetry. The torsional shielding electrodes and the fixed sensing electrodes are fabricated on the same plane and interdigitally arranged. When the microsensor is exposed to a vertical electric field, charge will be induced on these two arrays of sensing electrodes.

According to Gauss’ Law, the quantity of the induced charge is proportional to the sensing area and the electric field strength, as shown in Formula (1).

(1) $$Q_{s}=\varepsilon_{0}E_{n}A$$

where *Q_s_* is the induced charge, *ε*_0_ is the permittivity of the free space, *E_n_* is the strength of the measured electric field, and *A* is the effective sensing area. When the microsensor performs in torsional state, the quantity of the induced charge on the sensing electrodes array changes with the torsional angle of the shielding electrodes. When the torsional shielding electrodes are in their uppermost position, a majority of the electric field lines terminate on the torsional shielding electrodes rather than the sensing electrodes, thus the quantity of the induced charge is the least. When the torsional shielding electrodes rotate to their nethermost position, the situation is reversed. With the periodical vibration of the shielding electrodes, the induced charge on the sensing electrodes will also periodically change, generating alternate current *i_s_* as given by

(2) $$i_{s}=\frac{dQ_{s}}{dt}=\varepsilon_{0}E_{n}\frac{dA}{dt}$$

Notably, these two arrays of the sensing electrodes are differentially designed. The outputs of these two sensing electrodes arrays are identical but have 180° phase difference theoretically. These two outputs are connected to a differential amplifier to reduce common-mode noise with two identical trans-resistance amplifiers.

### 2.2. Structure Design

**Figure 2:**
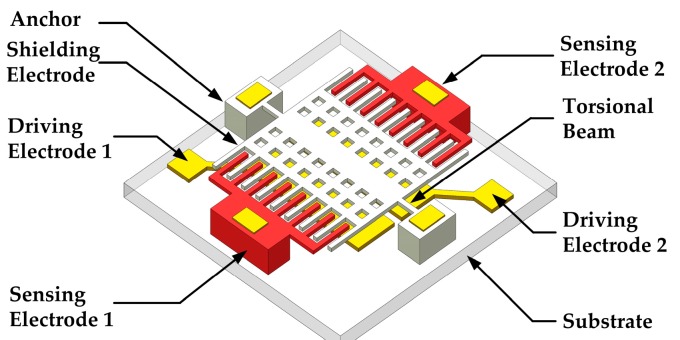
Schematic structure of the EFM.

As shown in Figure 2, the microsensor mainly consists of six parts, including the torsional beams, driving elements, shielding electrodes, sensing electrodes, anchors and the substrate.

We adopt electrostatic driving method as the actuation. Within the driving elements, to realize an out-of-plane torsion, two identical parallel plate structures are symmetrically arranged along the torsional axis, where the bottom driving electrodes are fabricated on the substrate, and the top driving electrodes are movable and connected to the shielding electrodes. Because the torsional angle is very small, it can be assumed that the electrostatic force is independent of the movable driving electrodes’ displacement. The electrostatic force is proportional to the square of the applied excitation signal. Herein, two excitation signals of *V_d_* + *V_a_*sin*ωt* and *V_d_* − *V_a_*sin*ωt* are applied to the driving electrode 1 and the driving electrode 2, respectively. Thus, the electrostatic force consists of three components related to *V_d_*^2^, *V_a_*^2^sin^2^*ωt* and ±*V_d_V_a_*sin*ωt*. However, the components related to *V_d_*^2^, *V_a_*^2^sin^2^*ωt* on the two driving elements are almost counteracted because of the symmetrical structure, and thus the component related to *V_d_V_a_*sin*ωt*, plays a major role in the electrostatic torque. Therefore, the driving force has the same frequency of the sinusoidal signals. Consequently, the torsional vibration frequency of the top driving electrodes and the shielding electrodes will be identical with the sinusoidal signals frequency, as well as the frequency of the sensed signal.

To increase the amplitude of the displacements, the shielding electrodes are placed far away from the torsional axis while the driving electrodes are near to the torsional axis. Thus, the end of the shielding electrodes could obtain the maximum displacement. This means that the shielding electrodes have larger displacement than that of the top driving electrodes under the same torsional angle, leading to the amplitude increasement.

In addition, for this resonant microsensor, increasing quality factor (*Q*) will also be helpful for the amplitude increasement. The gap between the movable and the fixed driving electrodes is small, and the *Q* is mainly affected by the squeeze film air damping in this torsional structure [22,23]. Taking that into account, holes are arranged on the movable driving electrodes to reduce the squeeze film air damping effect.

To enlarge the sensor’s response, the sensing electrodes and the shielding electrodes are coplanar and interdigital. In this sensing structure, all the electric field lines terminating on the top surface and the sidewalls of the sensing electrodes can be modulated, leading to the increasement of the effective sensing area. Meanwhile, two arrays of differential sensing electrodes are symmetrically arranged in different sides of the torsional axis to decrease the common-mode noise.

Owing to the special structure design of the torsional actuation, coplanar and interdigital electrodes, and differential sensing elements, this microsensor has advantages of high sensitivity, high efficiency of charge induction and low common-mode noise. The geometrical parameters are listed in Section 3.

## 3. Simulation and Optimization

### 3.1. Electric Field Distribution

**Figure 3:**
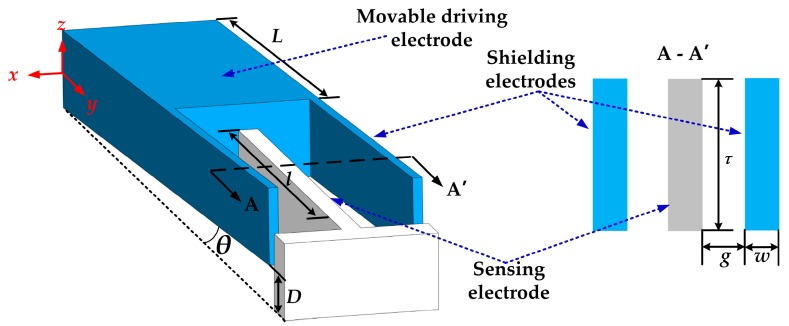
The schematic model of the sensitive structure for simulation. *x* is the torsional axis, *L* is the driving elements’ length, *l* is the length of the shielding and sensing electrodes, *τ* is the thickness of the sensitive structure, *g* is the gap between the sensing and shielding electrodes, *w* is the width of electrodes, *D* is the maximum displacement of the shielding electrodes, and *θ* is the torsional angle.

The simulations were conducted by finite element method (FEM) tool. The three-dimensional (3D) simulation model and the simplified two-dimensional (2D) simulation model of one unit of the sensitive structure are schematically shown in Figure 3. In essence, the shielding electrodes modulate the electric field distribution on the sensing electrodes by vertical displacement. Therefore, the simplified 2D simulation model is a typical section for the sensing unit, and the rules of the electric field distribution of this model can represent that of the whole sensitive structure.

Since the sensing electrodes and the shielding electrodes have the same electric potential with the ground, the measured electric field will distort on the electrodes, and the induced charge will be redistributed.

**Figure 4:**
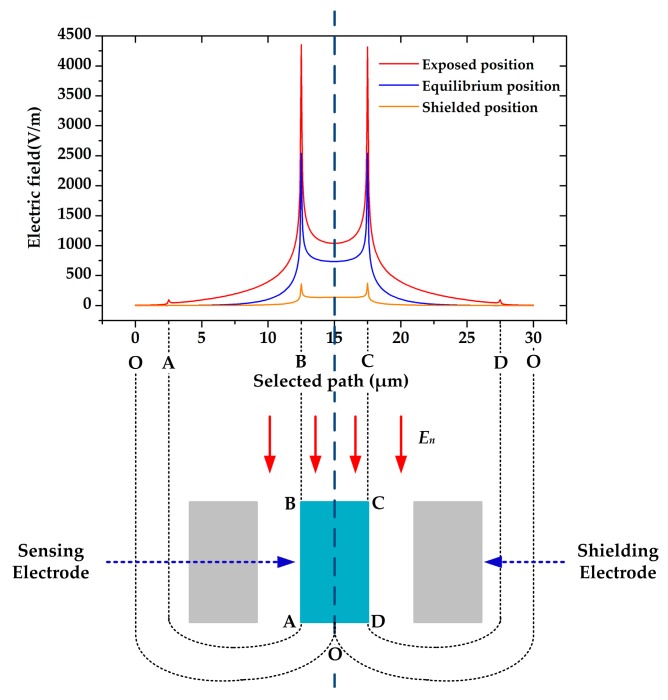
The simulation of electric field distribution on the sensing electrode when the sensing electrodes are in the exposed position, the equilibrium position and the shielded position, respectively.

For quantitative comparison, we simulated the electric field distribution on the sensing electrodes by applying an electric field of 1 kV/m. The simulation result is shown in Figure 4, where the clockwise path of the electrodes edge is selected as *x* axis, and the midpoint O of the bottom line is selected as the start and the end point. According to the simulation result, most components of the electric field terminate on the upside of the sensing electrodes, especially on the top surface. Furthermore, it is illustrated that the out-of-plane shielding method can change the electric field terminating on the sidewalls as well as on the top surface. We calculated the quantity of the induced charge by line integral, and the result shows that

(3) $$Q_{+}:Q_{0}:Q_{-}=129:64:10$$

where *Q*_+_, *Q*_0_, and *Q*_−_ are the quantity of the induced charge on the sensing electrode when the sensing electrodes are in the exposed, equilibrium and shielded positions, respectively. Compared with previous works, the efficiency of charge induction for EFM in this paper has been improved to a large extent.

### 3.2. Parameter Optimization

As the amplitude of the induced current is proportional to the amplitude of the induced charge variation on the sensing electrodes, we have optimized the structure parameters to get maximum output, with consideration of limited rules and fabrication capability. The key parameters need to be optimized are marked in Figure 3. To classify, the microsensors will be fabricated with an SOI wafer whose device silicon thickness is 25 µm, as introduced in Section 5. In the design, the sensitive structure thickness is 10 µm, the gap between the sensitive structure and the substrate is 15 µm, the length of the driving elements is 500 µm, the width of the driving elements is 1900 µm, torsional beam length is 100 µm, torsional beam width is 20 µm, and the electrodes length is 500 µm.

**Figure 5:**
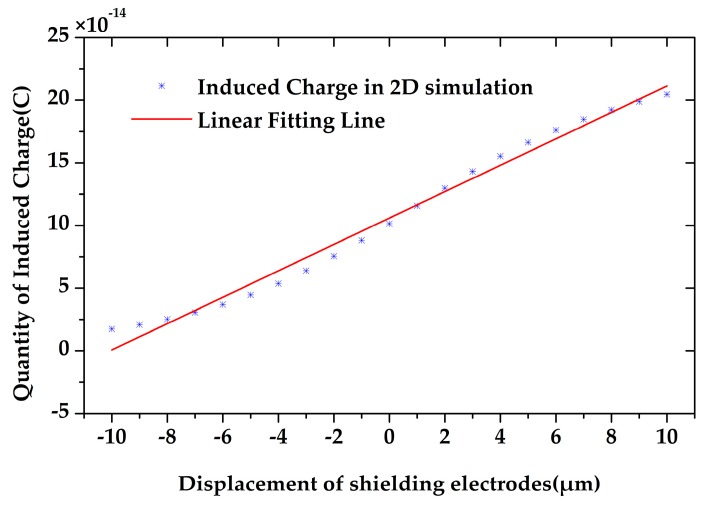
The induced charge on the sensing electrodes is proportional to the displacement of the shielding electrodes.

The quantity of the induced charge on the sensing electrodes is highly related to their positions. To observe the induced charge variation during the microsensor’s working time, we simulated the quantity of the induced charge with respect to different displacement of the shielding electrodes from −10 to 10 µm by 2D simulation. As shown in Figure 5, the quantity of the induced charge changes proportionally to the displacement of the shielding electrodes, which means that the output current will also be proportional to the velocity of the shielding electrodes. Meanwhile, the amplitude of the induced current will also be proportional to the maximum displacement of the shielding electrodes.

**Figure 6:**
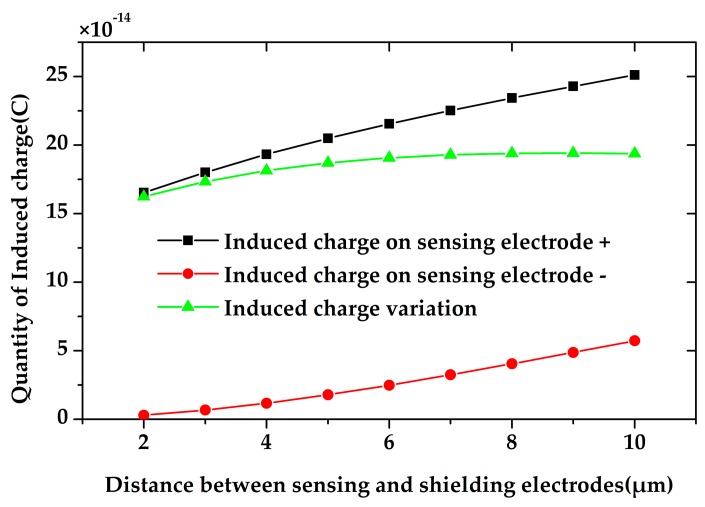
The simulation of the induced charge on the sensing electrodes versus the distance between the electrodes. The induced charge variation does not increase any more when the distance is larger than 5 µm.

Besides, the distance between the sensing electrodes and the shielding electrodes also has a large impact on the charge induction on the sensing electrodes. The induced charge on the sensing electrodes with respect to different distances between the sensing electrodes and the shielding electrodes was simulated. The result is shown in Figure 6, where the sensing electrode + is exposed and the sensing electrode—is shielded. It shows that, with the increasement of the distance, the induced charge on the two sensing electrodes both increase, and the induced charge variation increases by 25% as the distance changes from 2 µm to 5 µm. However, the induced charge variation will not increase any more when the distance is larger than 5 µm. Therefore, the distance between the sensing electrodes and the shielding electrodes is set to be 5 µm.

**Figure 7:**
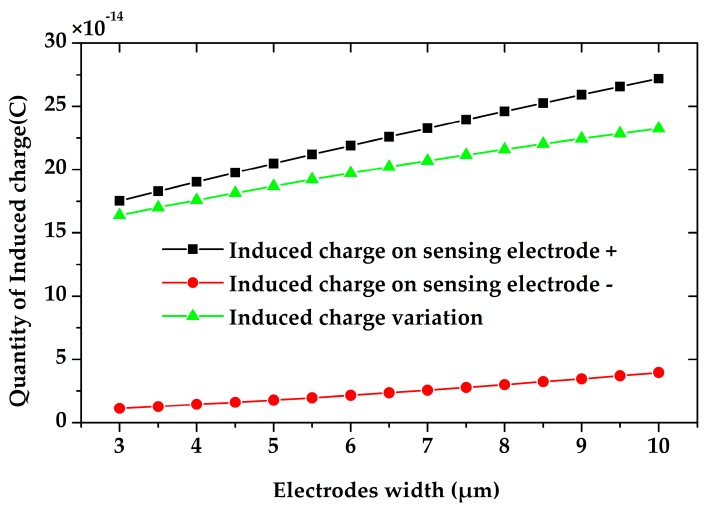
The simulated induced charge on the sensing electrodes versus the electrodes width. Wider electrodes lead to more induced charge and larger induced charge variation.

We also simulated the sensing electrodes width’s impact on the charge induction. As shown in Figure 7, the wider the electrodes are, the more charge will be induced on the sensing electrodes. The induced charge variation almost increases linearly to the electrodes width.

**Figure 8:**
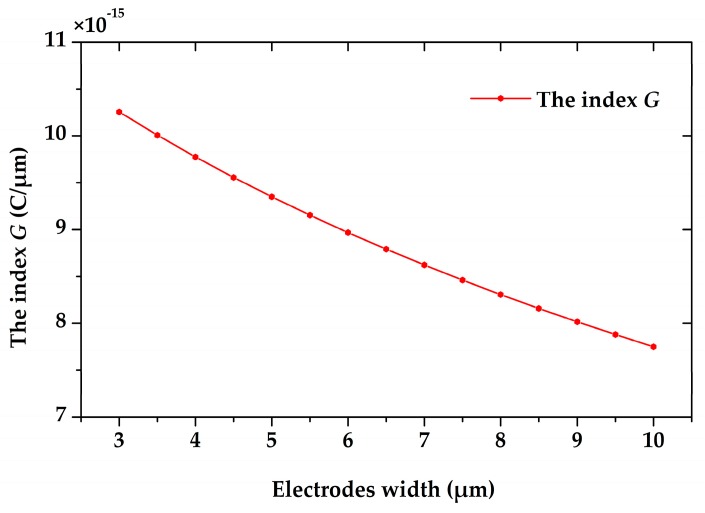
Simulation of the index *G* versus the electrodes width, as the index *G* is defined in Formula (4). The narrower the electrodes are, the larger the index *G* is, as well as the higher the capability is.

Nevertheless, with the limitation of area, the wider the electrodes are, the smaller the quantity of electrodes will be. Hence, we defined a new index *G* to characterize its capability of charge induction. The expression of the index *G* is shown as below

(4) $$G=\frac{Q_{sense}}{2w+2g}$$

where *Q_sense_* is the amplitude of the induced charge variation on one sensing electrode. With constant electrode length and limited sensing area, the larger the index *G* is, the higher capability the microsensor has. Figure 8 shows the change rule of the index *G* versus the electrodes widths, and the result indicates that narrower electrodes will lead to higher capability of charge induction for the microsensor. In fact, the width of the electrodes is limited by the fabrication process. In our laboratory, for long suspending beams, the limitation of the electrodes’ width is 5 µm, so we set the electrodes width to be 5 µm.

**Figure 9:**
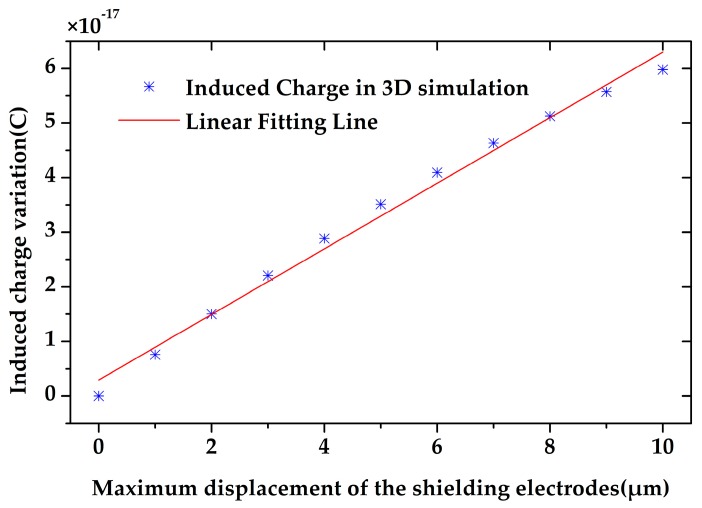
The induced charge variation on the sensing electrodes is proportional to the maximum displacement of the shielding electrodes.

Using the optimal geometrical parameters, the induced charge variation on single sensing electrode of the 3D model was simulated. The simulation result proved that the induced charge variation is proportional to the maximum displacement of the shielding electrodes, as shown in Figure 9. Since the torsional angle is very small, the maximum displacement (the displacement of the shielding electrodes’ end) is proportional to the torsional angle. Therefore, the induced charge variation is proportional to the torsional angle. In addition, the induced charge variation on single electrode is 5.98 × 10^−17^ C while the maximum displacement of shielding electrodes is 10 µm.

According to the simulation results above and the fabrication process limitation, the optimized parameters are listed in Table 1.

### 3.3. Resonant Mode Simulation

This microsensor is designed to work at its torsional resonant frequency to get a large vibration amplitude under low driving voltages. Therefore, the resonant frequency of this movable structure was simulated by FEM tools.

**Figure 10:**
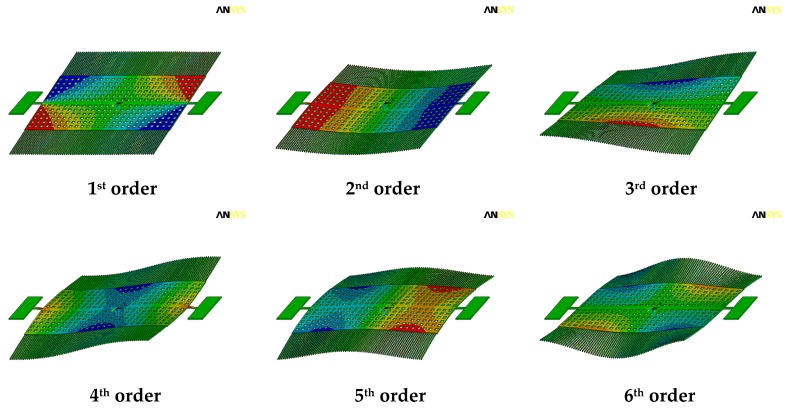
The first six orders resonant modes of the EFM extracted by simulation.

Referring to the realistic working status, the anchors are fixed in all degrees of freedom in the simulation. The first six orders resonant modes were extracted. As shown in Figure 10, the frequencies of the first six orders resonant modes are 5358 Hz, 10,653 Hz, 23,253 Hz, 28,794 Hz, 39,610 Hz and 45,004 Hz, respectively. The torsional resonant mode is the first order among them. The torsional resonant frequency of 5358 Hz is far away from the second order resonant frequency of 10,653 Hz. Thus, the other resonant modes would hardly be excited during the microsensor’s operation, and the interference between the working mode and the other modes will be reduced. Herein, the simulation was conducted without the consideration of the air damping and the negative electrostatic stiffness caused by the dc offset voltage in the actuation.

## 4. Analysis of Charge Induction Efficiency

For the microsensor, the efficiency of charge induction *C_cie_* represents its capability to transduce the strength of the electric field into an output current, and it can be defined as

(5) $$C_{cie}=\frac{di_{s0}}{dE_{n}}$$

where *i_s_*_0_ is the amplitude of the induced current.

**Figure 11:**
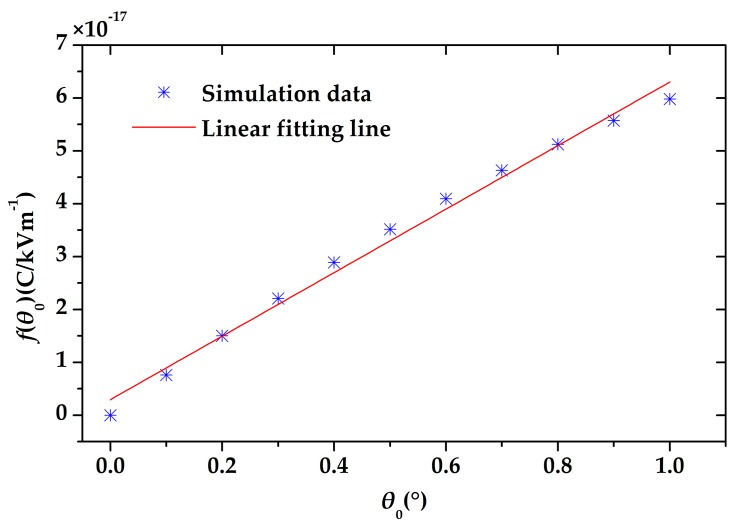
The amplitude of the induced charge variation per kV/m *f*(*θ*_0_) is proportional to the amplitude of the torsional angle *θ*_0_.

As the movable structure is actuated harmonically, the quantity of the induced charge on the sensing electrode can be expressed as

(6) $$Q(t)=Q_{0}\sin\omega t$$

where *Q*_0_ is the amplitude of the induced charge variation on the sensing electrodes, *ω* is the frequency of the ac component of the driving signal, and *Q*_0_ is proportional to the strength of the measured electric field *E_n_* given as below

(7) $$Q_{0}=f(\theta_{0})E_{n}$$

where *θ*_0_ is the amplitude of the torsional angle, *f*(*θ*_0_) is the amplitude of the induced charge variation under the electric field of 1 kV/m. We also simulated *f*(*θ*_0_) under different torsional angles *θ*_0_. According to the simulation result shown in Figure 11, it proves that *f*(*θ*_0_) is nearly linear to the torsional angle *θ*_0_, as given by

(8) $$f(\theta_{0})=k\theta_{0}$$

where *k* is a certain coefficient related to the structure parameters. According to the above formulas, the induced current is

(9) $$i_{s}=\frac{dQ(t)}{dt}=k\theta_{0}\omega E_{n}\cos\omega t$$

Finally, the efficiency of charge induction *C_cie_* can be expressed as

(10) $$C_{cie}=k\theta_{0}\omega$$

According to Formula (10), we can improve the efficiency of charge induction through three methods as below.

(a) Increasing *k*. The optimization of the sensitive structure design will be helpful, such as adopting coplanar interdigital electrodes and out-of-plane vibration.
(b) Increasing *θ*_0_. The amplitude of the torsional angle can be raised by raising the driving voltages. However, the torsional angle is limited because of the pull-in effect.
(c) Increasing excitation frequency *ω*. However, for this resonant microsensor, it is best to work at resonant frequency for gaining the largest torsional angle *θ*_0_.

## 5. Fabrication

We completed the fabrication of the microsensor in our laboratory by breaking through some key process, and the fabrication process is based on the anodic bonding and SOI technologies.

Because the thickness of the sensitive structure is as thin as 10 µm, to get a high precision of the sensitive structure’s thickness, we decided to fabricate the sensitive structure on the device silicon of an SOI wafer. The device silicon thickness is 25 µm, and a trench will be fabricated on the SOI device silicon to form a 15 µm height difference as the gap between the sensitive structure and the substrate.

**Figure 12:**
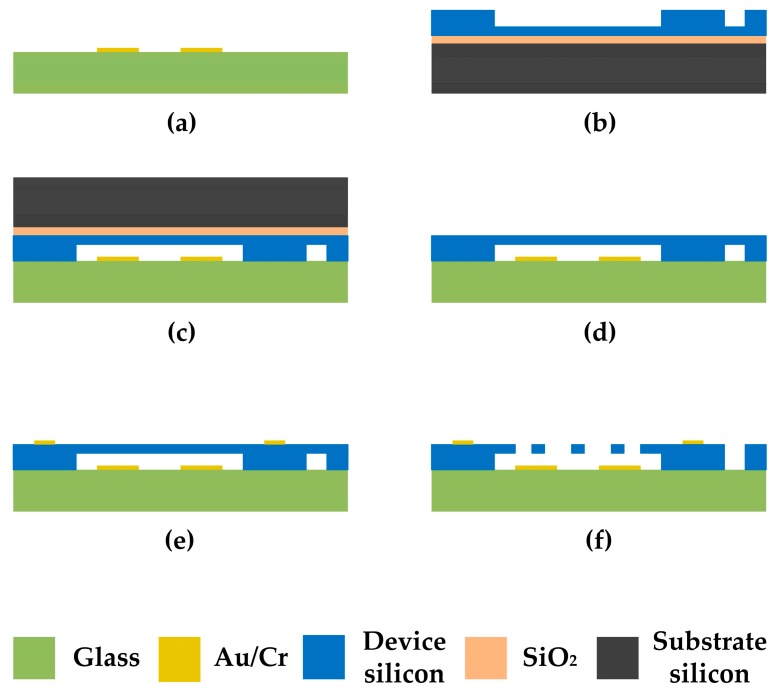
The main steps of the fabrication process.

The main fabrication process illustrated in Figure 12a–f are described as below:

(a) Metal layer is deposited on the glass substrate as driving electrodes.
(b) Deep reactive ion etching (DRIE) is used to form a cavity on the device silicon of an SOI wafer.
(c) The anodic bonding of the SOI wafer and the glass substrate is conducted.
(d) The substrate silicon and SiO_2_ layers are removed completely.
(e) Metal pads are deposited on the device silicon layer as an ohmic contact.
(f) DRIE is used to pattern the device structure, with the shielding electrodes and the sensing electrodes included.

As designed, the bottom driving electrodes are deposited on the glass substrate. The anodic bonding is employed to the SOI wafer and the glass substrate to realize firm connection of these two parts. High quality of the anodic bonding is necessary for successful removing of the substrate silicon, because the substrate silicon will be etched in KOH solution in the next steps.

In addition, the movable structures are fabricated by DRIE process. Due to the tension of the liquid, the narrow electrodes are prone to be adhered with each other. To avoid of the electrodes adhesion, fewer wet process steps should be conducted, especially after releasing the movable structure.

**Figure 13:**
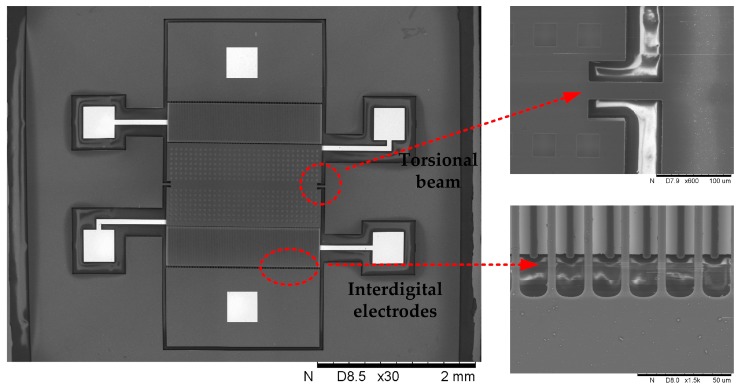
Scanning electron microscope (SEM) photos of the fabricated microsensor.

Scanning electron microscope (SEM) photos of the fabricated microsensor are shown in Figure 13. The dimension of the whole EFM is 6 mm × 6 mm. The device is complete and clean, and the key dimension of the structure is in accordance with the design.

## 6. Experiments

**Figure 14:**
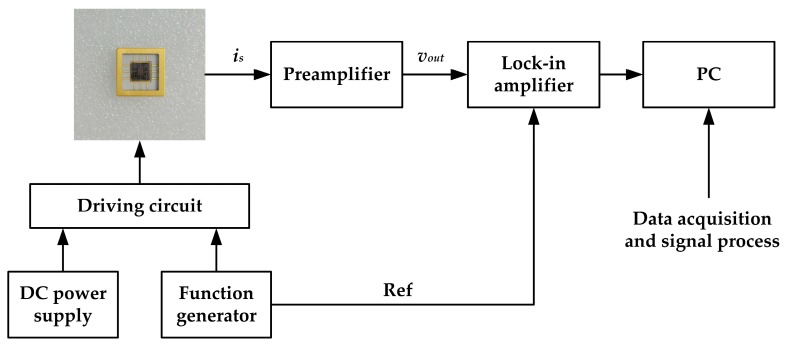
The lock-in amplifier-based system used to characterize the EFM.

The experiments were conducted using our self-developed calibration system under atmospheric pressure at room temperature. Figure 14 shows the schematic diagram of the calibration system. In the system, a sinusoidal signal was generated by the function generator. Firstly, the dc power supply and function generator were used to drive the microsensor. Then the output currents of the microsensor would be converted into a voltage by the preamplifier with a trans-resistance of 100 MΩ. Finally, a lock-in amplifier was used to extract the amplitude of the output voltage.

**Figure 15:**
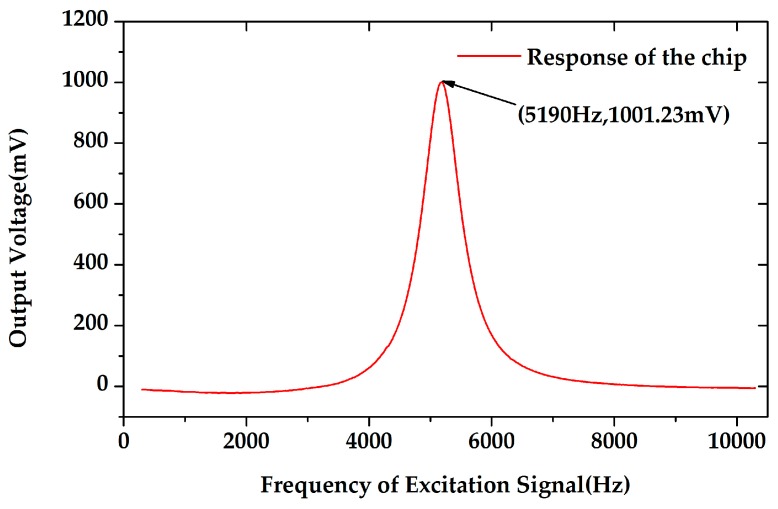
The amplitude-frequency response of the microsensor. The resonant frequency of the microsensor was 5190 Hz.

As the microsensor is designed to work in resonant mode, the amplitude-frequency response was tested with the frequency range of 300–10,300 Hz. As shown in Figure 15, the tested resonant frequency of the EFM was 5190 Hz, and the *Q* was calculated to be 10.81. The tested resonant frequency was in good accordance with the simulation result of 5358 Hz. The discrepancy was related to the fabrication process variation and the simulation deviation.

**Figure 16:**
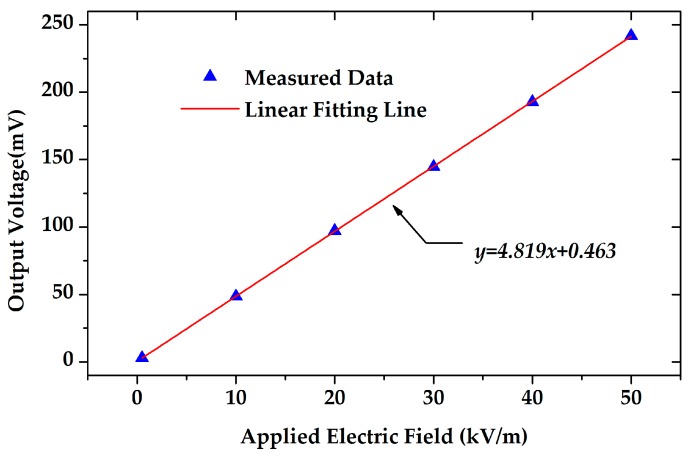
The response of the microsensor versus the applied electric field. The linearity was 0.15%.

As mentioned above, the proposed EFM has high sensitivity in limited sensing area. The response characteristics of the microsensor was tested with an electrostatic field range of 0–50 kV/m, and the experimental result is shown in Figure 16. The EFM had a good linearity of 0.15%, and the sensitivity of the EFM reached 4.82 mV/(kV/m). Since the trans-resistance of the preamplifier was 100 MΩ, the efficiency of charge induction was calculated to be 48.19 pA/(kV/m).

In addition, according to the simulation results of the induced charge variation and the resonant frequency of this EFM, the sensitivity has been calculated with a trans-resistance of 100 MΩ. The comparison of the experimental and simulation results of this EFM is shown in Table 2. The tested sensitivity was in good accordance with the simulation result 1, which means that the maximum displacement of the shielding electrodes was about 7 µm. Consequently, the maximum displacement of the driving electrodes was about 3.5 µm, according to structure design illustrated in Section 2. Affected by the squeeze film damping, in open air, the shielding electrodes did not achieve a maximum displacement of 10 µm as simulated.

Furthermore, a comparison was made between recently reported available devices as shown in Table 3. As introduced in Section 2, the proposed EFM has a special structure with a torsional shutter and coplanar interdigital electrodes. Thus, all the electric field distribution on the top surface and sidewalls of sensing electrodes can be modulated, resulting in effective sensitive area increase. Compared with the previous available EFMs, the proposed EFM sensitivity has been improved by at least one order of magnitude, and it has been proven that the proposed sensitive structure has higher efficiency of charge induction.

**Figure 17:**
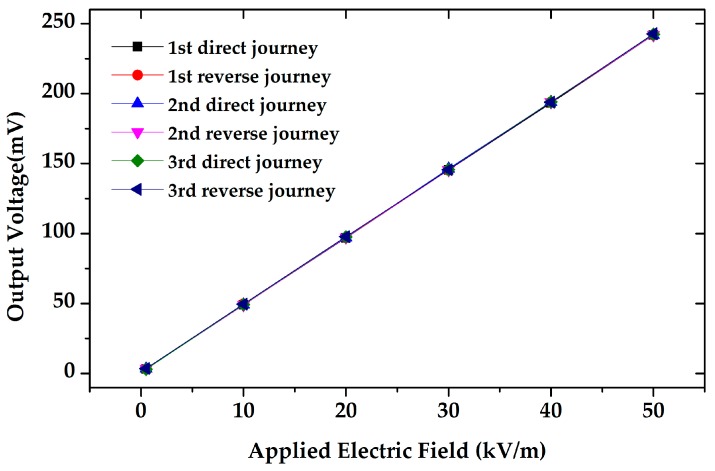
The response of the microsensor in the three roundtrip measurement. The uncertainty of the microsensor was 0.38%.

For further characterization, the microsensor was tested in the three roundtrip measurements under an electrostatic field range of 0–50 kV/m, and the experimental result is shown in Figure 17. The uncertainty of the EFM was calculated to be 0.38%, which indicated that the microsensor has very high precision for electrostatic field measurement.

## 7. Conclusions

A high sensitivity EFM based on torsional resonance is proposed and developed. A torsional shutter composed of shielding electrodes and torsional beams is adopted in the EFM. The movable shielding electrodes and the fixed sensing electrodes are coplanar and interdigital. As proven by simulations, the sensitivity and efficiency of charge induction are greatly improved by combination of the torsional shutter and the coplanar interdigital sensing structure. The optimization of structure parameters was conducted to improve its efficiency of charge induction further. A micromachining fabrication process was developed to fabricate the EFM, which was based on the anodic bonding and SOI technologies. Experimental results showed that a good linearity of 0.15% was achieved under an electrostatic field range of 0–50 kV/m, and the uncertainty was below 0.38% in the three roundtrip measurements. A high sensitivity of 4.82 mV/(kV/m) was achieved with the trans-resistance of 100 MΩ, which was improved by at least one order of magnitude compared with previously reported EFMs. The efficiency of charge induction for this microsensor reached 48.19 pA/(kV/m). Thus, the proposed microsensor has demonstrated great potential for future electric field measuring applications.

## Figures and Tables

**Table 1:** The optimal parameters of the EFM.

Symbol	Structural Parameters	Value
*τ*	thickness of the sensitive structure	10 µm
*d*	distance between the sensitive structure and substrate	15 µm
*W*	width of the driving elements	1900 µm
*L*	length of the driving elements	500 µm
*w*_t_	width of the torsional beam	20 µm
*l*_t_	length of the torsional beam	100 µm
*w*	width of the electrodes	5 µm
*l*	length of the electrodes	500 µm
*g*	gap between the electrodes	5 µm
*N*_e_	number of the sensing electrodes	94 × 2
*l*_h_	side length of the square holes	30 µm
*N*_h_	number of the square holes	217 × 2
*Q*_e_	induced charge variation on single electrode (with the shielding electrodes’ maximum displacement of 10 µm)	5.98 × 10^−17^ C

**Table 2:** Comparison between experimental and simulation results of this EFM.

Data Resources	Conditions	Sensitivity
Experimental result	Tested in open air	4.82 mV/(kV/m)
Simulation result 1	The maximum displacement of shielding electrodes is 7 µm	4.67 mV/(kV/m)
Simulation result 2	The maximum displacement of shielding electrodes is 8 µm	5.16 mV/(kV/m)
Simulation result 3	The maximum displacement of shielding electrodes is 10 µm	6.02 mV/(kV/m)

**Table 3:** Sensitivity comparison of reported EFMs.

EFMs	Year	Sensitivity
MEMS field mill [16]	2001	40 nV/(kV/m)
EFM using thermal actuators [17]	2007	0.1 mV/(kV/m)
Thermally driven resonant EFM [12]	2008	0.098 mV/(kV/m)
Micromachined EFM [18]	2008	0.15 mV/(kV/m)
SOI EFM [15]	2011	0.2 mV/(kV/m)
Electrostatic biased EFM [19]	2015	0.84 mV/(kV/m)
EFM in this paper	2017	4.82 mV/(kV/m)
